# Pharmacologic Management of Non‐Traumatic Dental Conditions in US Emergency Departments, 2018–2022

**DOI:** 10.1111/jphd.12668

**Published:** 2025-04-11

**Authors:** Leah I. Leinbach, Xiaobai Li, Timothy Iafolla, Hosam Alraqiq

**Affiliations:** ^1^ National Institute of Dental & Craniofacial Research National Institutes of Health Bethesda Maryland USA; ^2^ National Institutes of Health Clinical Center National Institutes of Health Bethesda Maryland USA

**Keywords:** antibiotics, emergency department visits, opioid analgesics, oral health

## Abstract

**Objective:**

This study examines opioid and antibiotic prescribing by United States emergency departments (EDs) for non‐traumatic dental conditions (NTDCs) between 2018 and 2022.

**Methods:**

This is a secondary analysis of nationally representative ED visits using the National Hospital Ambulatory Medical Care Survey (NHAMCS) with an NTDC as the primary discharge diagnosis. Descriptive statistics and odds ratios using chi‐squared testing and multivariable logistic regression were used to examine analgesic and antibiotic prescriptions, as well as patient, visit, and hospital characteristics.

**Results:**

There were 1,838,729 weighted ED visits for NTDCs between 2018 and 2022, 1.3% of all visits. Findings demonstrate a continued decline in NTDC visits resulting in an opioid, with an increase in those with non‐opioids. Overall, 25% of NTDC visits included an opioid analgesic in 2022, compared to 33% in 2018. The proportion of visits with non‐opioid analgesics increased over the study period; nearly 60% of NTDCs seen in 2020 included a non‐opioid analgesic. Overall, 63% included an antibiotic, with the highest proportion observed in 2020 (70%). No increase in the proportion of ED visits for NTDCs was seen between the pandemic years (2020–2022) and the pre‐pandemic period (2018–2019).

**Conclusions:**

Antibiotics and non‐opioid analgesics were a common approach used by ED providers during the pandemic years. Opioid prescriptions for NTDCs declined between 2018 and 2022, while antibiotic prescriptions remained roughly stable. Reducing avoidable opioid and antibiotic prescriptions, and more broadly ED visits for NTDCs, requires a comprehensive approach.

## 1 Introduction

Non‐traumatic dental conditions (NTDCs) encompass a wide range of disorders involving the teeth and surrounding structures, including periapical abscess, pulpitis, periodontitis, and dental caries [1]. Most NTDCs are chronic and best managed in outpatient oral healthcare settings [2] with restorative therapy, endodontic therapy, or oral surgery. When access to regular care is inaccessible or unavailable, emergency departments (EDs) can serve as a safety net. Treatment for NTDCs in EDs often involves palliation using analgesics, antibiotics, or both, until treatment by an oral healthcare provider outside of the hospital system can be obtained.

NTDCs have been among the most common reasons for opioid prescription in the ED [3, 4]. Between 2015 and 2017, nearly 40% of ED visits for oral health problems included an opioid prescription, almost five times higher than visits for other reasons [5]. Antibiotics are also commonly prescribed for NTDCs [6] despite inconclusive evidence in support of clinical benefit, particularly among patients presenting without obvious signs or symptoms of infection [7]. Adverse consequences of antibiotic use, such as allergic reactions and antibiotic resistance, can occur. Opioid analgesics have the potential for misuse and diversion [8, 9].

The significant and sustained disruptions to the United States healthcare system consequent to the COVID‐19 pandemic changed the ways in which healthcare services could be accessed. In the years preceding the COVID‐19 pandemic, there was a sustained decline in opioids prescribed by the United States healthcare system [10]. There were also continued efforts to minimize unnecessary exposure to antibiotics in an effort to reduce resistance and other unintended consequences [11]. In the first year of the pandemic (2020), closures to outpatient dental offices limited the availability and type of care delivered. While early pandemic data from a single state showed no increase in ED visits for oral health problems [9], to our knowledge, no national‐level assessment of ED visits for NTDCs during the pandemic years has been completed to date. Continued pandemic‐related disruptions in outpatient oral health care may have led to increased ED visits for NTDCs, higher severity of NTDCs, and greater reliance on medical management (i.e., more opioid and antibiotic prescriptions). We analyze nationally representative data to better understand pharmacologic management of NTDCs by U.S. EDs from 2018 to 2022.

## 2 Methods

### 2.1 Study Design and Data Source

This is a cross‐sectional analysis of publicly available emergency department visit data from 2018 to 2022 National Hospital Ambulatory Medical Care Survey (NHAMCS). NHAMCS is an annual survey of EDs in all 50 states and the District of Columbia supported by the National Center for Health Statistics and the United States Census Bureau. NHAMCS is administered using a three‐stage probability design at regional, hospital, and patient visit levels, with population ratio adjustments based on annual patient visit volumes [12]. Variables with a nonresponse rate greater than 5% were imputed using a methodology described in the NHAMCS codebook [12]. Because of the public availability of the dataset, this study was excluded from Institutional Review Board approval.

### 2.2 Non‐Traumatic Dental Conditions and Medications

Emergency department visits for non‐traumatic dental conditions (NTDCs) were defined according to the Association of State and Territorial Dental Directors (ASTDD) International Classification of Disease Version 10 (ICD‐10) codes for NTDCs [1] (Table S1). Diagnostic codes were further grouped into the broad disease categories: dental caries, pulpal and periapical disorders, disorders of the teeth and surrounding structures, disorders of the tongue, cellulitis, periodontal disease, and other (i.e., salivary gland disorders, TMDs etc.) (Table S2). Medications were defined based on Cerner Multum's therapeutic classifications and drug component category codes [12] (Table S3).

### 2.3 Sociodemographic, Clinical and Hospital Characteristics

Patient sociodemographic characteristics included age, sex, race, ethnicity, and primary expected medical payor. Clinical characteristics included the number and type of chronic conditions (other than the primary NTDC diagnosis), patient‐reported pain, and objective fever. Provider type, diagnostic tests ordered (i.e., CBC and imaging studies), length of ED visit, disposition (i.e., admitted to hospital versus discharged from ED) and visit year were reported. Hospital characteristics included region, rurality, and teaching hospital status, defined as the presence or absence of an emergency medicine residency.

### 2.4 Statistical Methods

Data were weighed per NHAMCS guidelines for national estimates, unless otherwise specified [12]. Categorical variables were reported as frequencies and percentages, continuous variables as means, medians, and ranges. Differences by study year were assessed using chi‐squared testing. Multivariable logistic regression identified significant factors among prescriptions and patient demographics, chronic conditions, primary payor, hospital region, rurality, teaching status, provider type, disposition, and visit year, accounting for complex survey design. A *p*‐value < 0.05 was considered significant. Analyses were performed using SAS 9.4 (Cary, NC) and GraphPad Prism 10 (Boston, MA).

## 3 Results

### 3.1 Characteristics of NTDC Visits

There were 86,864 total ED visits between 2018 and 2022 (weighted: 139,581,179); 1.3% of visits (SE: 0.06) were for NTDCs (Table 1). Among NTDC visits, diseases of the teeth and surrounding structures were the most common primary diagnoses (37.5%), followed by pulpal and periapical disorders (25.2%) (Table 1). Although there was a drop in total ED visits in 2020, the proportion of visits for NTDCs remained roughly stable across all years (Table 2). No association between visit type and visit year was observed (*p* = 0.59).

**TABLE 1:** Characteristics of emergency department visits for non‐traumatic dental conditions (United States, 2018–2022).

Characteristic	Unweighted Freq. *n* = 1129	Weighted Freq. *n* = 1838729	% (SE)^a^
Age			
< 18	187	316879	17.2 (1.9)
18–39	605	998913	54.3 (2.2)
40–64	279	436568	23.7 (1.5)
65+	58	86369	4.7 (0.9)
Sex			
Male	549	872634	47.5 (1.9)
Female	580	966096	52.5 (1.9)
Race			
African American/Black	375	592893	32.2 (2.4)
White	732	1214174	66.0 (2.4)
Other	22	31661	1.7 (0.5)
Ethnicity			
Hispanic or Latino	162	267524	14.5 (1.9)
Non‐Hispanic or Latino	967	1571205	85.5 (1.9)
Primary Payor			
Private	216	370334	20.1 (1.6)
Medicare	85	130878	7.1 (1.2)
Medicaid	538	891145	48.5 (3.3)
Self‐Pay	164	242249	13.2 (1.5)
Other or unknown	126	204124	11.1 (2.4)
Provider type			
Physician	862	1348293	73.3 (2.7)
Non‐physician	267	490437	26.7 (2.7)
Length of visit (min)			
Mean	118.5	—	—
Median	72.0	—	—
Range	0‐2033	—	—
Hospital region			
Northeast	205	279461	15.2 (2.6)
Midwest	294	412531	22.4 (3.1)
West	231	409994	22.3 (2.8)
South	399	736743	40.1 (4.6)
Hospital rurality			
Urban	961	1595544	86.8 (3.8)
Rural	168	243185	13.2 (3.8)
Hospital teaching status			
Academic	399	554345	30.2 (3.6)
Community	669	1197882	65.1 (3.5)
Blank or unknown	61	86503	4.7 (1.1)
Number of chronic conditions^b^			
None	689	1174863	63.9 (2.0)
One	222	318861	17.3 (1.3)
Two or more	218	345005	18.8 (1.8)
Type of chronic conditions^c^			
Asthma	107	152646	8.3 (1.2)
Depression	116	196861	10.7 (1.4)
Diabetes^d^	79	125065	6.8 (1.1)
Hypertension	183	266474	14.5 (1.2)
Obesity	73	117630	6.4 (1.6)
Substance use disorder	84	130266	7.1 (1.3)
Primary diagnosis			
Dental caries	141	244969	13.3 (1.5)
Pulpal and periapical disorders	281	463097	25.2 (1.6)
Disorders of teeth/hard tissue	429	688896	37.5 (2.1)
Disorders of the tongue	60	96052	5.2 (0.9)
Cellulitis	19	23284	1.3 (0.4)
Periodontal disease	24	42300	2.3 (0.8)
Other	175	280132	15.2 (1.4)
Severe pain^e^			
Yes	451	733244	39.9 (3.0)
No	678	1105485	60.2 (3.0)
Fever^f^			
Yes	19	41568	2.3 (0.69)
No	1110	1797161	97.7 (0.69)
Diagnostic tests			
CBC	120	205968	11.2 (1.2)
Imaging	126	200272	10.9 (1.2)
Disposition			
Admitted	17	32514	1.8 (0.6)
Discharged	1112	1806216	98.2 (0.6)

^a^ Weighted percentage and standard error.

^b^ NHAMCS tracks 22 common chronic conditions: Alcohol use disorder, Alzheimer's disease, asthma, cancer, cerebrovascular disease, chronic kidney disease, chronic obstructive pulmonary disease, congestive heart failure, coronary artery disease, depression, diabetes (Type 1, Type 2, and unspecified), end‐stage renal disease, history of pulmonary embolism, human immunodeficiency virus, hyperlipidemia, hypertension, obesity, obstructive sleep apnea, osteoporosis, and substance use disorder.

^c^ Chronic conditions documented in 5% or more of NTDC visits.

^d^ Includes Type 1 diabetes, Type 2 diabetes, and unspecified diabetes.

^e^ Subjective pain was expressed on a 0–10 scale with 0 indicating no pain and 10 the worst pain imaginable. Responses of 7–10 on this scale were considered severe pain.

^f^ Fever was considered to be any objective temperature at or above 100° Fahrenheit.

**TABLE 2:** Non‐traumatic dental conditions as a proportion of total emergency department visits (United States, 2018–2022).

	NTDCs			Non‐NTDCs			*p* ^b^
Visit year	Unweighted	Weighted	% (SE)^a^	Unweighted	Weighted	% (SE)	
2018	290	374748	1.4 (0.1)	20001	25620040	98.5 (0.1)	0.59
2019	253	418399	1.3 (0.1)	19228	29711559	98.6 (0.1)	
2020	197	323823	1.3 (0.2)	14663	25935497	98.7 (0.2)	
2021	196	342130	1.2 (0.1)	16011	27614162	98.8 (0.1)	
2022	193	379629	1.2 (0.1)	15832	30699920	98.8 (0.1)	
2018–2022	1129	1838729	1.3 (0.06)	85735	139581179	98.7 (0.06)	

^a^ Proportion of total ED visits for respective year.

^b^ Association between visit type and visit year assessed using chi‐squared testing.

Most visits were made by working‐age adults, with 54.3% between 18 and 39 years of age, evenly distributed between men and women (Table 1). A majority of visits were by patients who were white 5(66.0%) and non‐Hispanic (85.4%). Medicaid was the primary expected payor for 48.5% of visits, followed by private insurance (20.1%) and uninsured/self‐pay (13.2%) (Table 1). Most visits were staffed by physicians (73.3%) and occurred at urban (86.8%), non‐teaching hospitals (65.1%), and predominantly in the South (40.1%). Among teaching hospitals where NTDCs were seen, a higher proportion were in the South; no differences in provider type, reported pain, or fever were observed (Table S5).

About one‐third (36.1%) of NTDC visits were made by patients with one or more chronic conditions, 18.8% by patients with two or more, with hypertension, depression, asthma, and substance use disorders being the most common co‐listed conditions (Table 1). 40% presented with a report of severe pain, and few (2.3%) had objective fever. A complete blood count (CBC) was ordered for 11.2% of visits, imaging studies for 10.9% of visits (most commonly a CT scan). Of patients with severe pain, the majority were in the 18–39 age range, had Medicaid, and had a diagnosis of a disorder of the teeth and surrounding structures (Table S6). The median length of visit was 72 min (Range: 0–2033 min).

Two percent of visits for NTDCs resulted in a hospital admission (Table 1). Most admissions (61%) were for people with one or more chronic conditions, with hypertension, obesity, and diabetes the most common chronic disease diagnoses (Table S4). Pulpal and periapical disorders were the most common primary diagnoses among patients admitted (62.4%, SD: 14.5), followed by cellulitis (18.4%, SD: 10.8). The median length of stay was 3 days (Range: 1–5 days).

### 3.2 Analgesic Prescriptions for NTDCs

64.2% of NTDC visits included some type of analgesic, roughly one‐third (29.8%) of which were an opioid or opioid combination analgesic, 45.9% a non‐opioid, and 11.5% an opioid and a non‐opioid (Table 3). Most opioids prescribed (62.6%, SE: 3.8) were opioid combination medications, and 37.4% (SE: 3.8) were opioid‐only formulations. Disorders of the teeth and surrounding structures were the most common diagnoses among visits for which an opioid was prescribed (43.4%, SD: 3.4), followed by pulpal and periapical disorders (29.4%, SD: 3.2) (Table 4). Of non‐opioids prescribed, most (88.7%, SE: 2.0) were non‐steroidal anti‐inflammatories (NSAIDs).

**TABLE 3:** Analgesics and antibiotics for NTDCs by visit year (United States, 2018–2022).

	Drug combination weighted Freq, % (SE)					
Visit year	Opioid	Non‐opioid	Opioid + non‐opioid	Antibiotic	Antibiotic + opioid	Antibiotic + non‐opioid
2018	124354, 33.2 (3.5)	148435, 39.6 (3.6)	40479, 10.8 (2.2)	244205, 65.1 (3.0)	100069, 36.7 (3.2)	118960, 31.7 (3.6)
2018–2019	*p* = 0.73	*p* = 0.25	*p* = 0.86	*p* = 0.18	*p* = 0.61	*p* = 0.71
2019	131521, 31.4 (3.8)	193648, 46.3 (4.2)	47307, 11.3 (2.3)	250196, 59.8 (3.3)	101406, 24.2 (3.4)	124964, 29.9 (3.5)
2019–2020	*p* = 0.97	*p* = 0.09	*p* = 0.28	*p* = 0.10	*p* = 0.28	*p* = 0.65
2020	102308, 31.6 (4.6)	189718, 58.6 (5.1)	50799, 15.7 (3.7)	225631, 69.7 (5.4)	77403, 23.9 (3.6)	145930, 45.1 (5.6)
2020–2021	*p* = 0.39	*p* = 0.06	*p* = 0.13	*p* = 0.46	*p* = 0.13	*p* = 0.13
2021	90946, 26.6 (3.8)	153097, 44.7 (5.2)	31439, 9.1 (2.2)	218705, 63.9 (5.3)	80752, 23.6 (4.0)	111027, 32.5 (5.2)
2021–2022	*p* = 0.71	*p* = 0.71	*p* = 0.64	*p* = 0.25	*p* = 0.64	*p* = 0.91
2022	96587, 25.4 (3.6)	159554, 42.0 (6.2)	41394, 10.9 (3.0)	212372, 55.9 (4.3)	89306, 23.5 (3.6)	119961, 31.6 (5.2)

*Note*: Chi‐squared testing used to assess differences in proportion of medications prescribed between specified visit year and previous year.

**TABLE 4:** Characteristics of United States emergency departments for visits NTDCs by medication type (2018–2022).

	Medication type weighted frequency, % (SE)					
Characteristic	Opioid *n* = 545716, 29.5 (2.0)	*p*	Antibiotic *n* = 1151110, 62.6 (2.2)	*p*	Antibiotic + opioid *n* = 448936, 24.4 (1.8)	*p*
Age						
< 18	13800, 2.5 (1.2)	<.0001	78164, 6.7 (1.2)	<.0001	9045, 2.0 (1.3)	<.0001
18–39	346902, 63.6 (3.9)		677130, 58.8 (2.4)		277051, 61.7 (4.3)	
40–64	166834, 30.6 (3.9)		346142, 30.1 (2.1)		145438, 31.4 (4.2)	
65+	18180, 3.3 (1.1)		49674, 4.3 (1.0)		17402, 3.9 (1.3)	
Sex						
Male	251104, 46.0 (3.2)	0.59	555914, 48.3 (2.0)	0.54	217935, 48.5 (3.6)	0.71
Female	294613, 54.0 (3.2)		595196, 51.7 (2.0)		231002, 51.5 (3.6)	
Race						
African						
American/Black	162594, 29.8 (4.0)	0.05	359429, 31.2 (3.0)	0.37	149908, 33.4 (4.2)	0.14
White	381475, 69.9 (3.9)		777166, 67.5 (3.0)		297845, 66.3 (4.2)	
Other	1650, 0.3 (0.2)		14514, 1.3 (0.6)		1183, 0.3 (0.2)	
Ethnicity						
Hispanic	62935, 11.5 (2.7)	0.19	151132, 13.1 (2.2)	0.21	53453, 11.9 (2.9)	0.30
Non‐Hispanic	482781, 88.5 (2.7)		999978, 86.9 (2.2)		395483, 88.1 (2.9)	
Primary payor						
Private	106333, 19.5 (3.1)	0.59	209298, 18.2 (2.1)	0.01	76143, 17.0 (3.3)	0.56
Medicare	32372, 5.9 (1.7)		85128, 7.4 (1.4)		30782, 6.9 (1.9)	
Medicaid	268402, 49.2 (5.0)		572127, 49.7 (4.0)		224840, 50.1 (5.4)	
Self‐pay	87413, 16.0 (2.5)		185188, 16.1 (2.0)		73613, 16.4 (3.2)	
Other or unknown	51197, 9.4 (2.7)		99368, 8.6 (1.7)		43558, 9.7 (2.9)	
Provider type						
Physician	396802, 72.7 (4.9)	0.87	843112, 73.2 (2.9)	0.94	333410, 74.3 (5.0)	0.81
Non‐physician	148914, 27.3 (4.9)		307998, 26.8 (2.9)		115527, 25.7 (5.0)	
Hospital region						
Northeast	54502, 10.0 (2.2)	0.05	141003, 12.3 (2.6)	0.15	46734, 10.4 (2.4)	0.11
Midwest	109751, 20.1 (4.7)		269104, 23.4 (3.9)		89995, 20.0 (4.9)	
West	151839, 27.8 (5.0)		257283, 22.4 (3.2)		122598, 27.3 (5.2)	
South	229624, 42.1 (6.2)		483690, 42.0 (5.2)		189609, 42.2 (6.8)	
Hospital rurality						
Urban	477472, 87.5 (4.1)	0.73	971225, 84.4 (4.5)	0.002	389452, 86.8 (4.6)	0.99
Rural	68245, 12.5 (4.1)		179884, 15.6 (4.5)		59484, 13.3 (4.6)	
Hospital teaching status						
Academic	410057, 75.1 (3.0)	<.0001	807788, 70.2 (3.3)	<.0001	336305, 74.9 (3.2)	<.0001
Community	105430, 19.3 (2.8)		266668, 23.2 (3.1)		82402, 18.4 (2.9)	
Blank or unknown	30230, 5.5 (1.7)		76653, 6.7 (1.4)		30230, 6.7 (2.0)	
Chronic conditions (#)^a^						
None	308738, 56.6 (3.8)	0.03	703392, 61.1 (2.9)	0.28	258200, 57.5 (4.2)	0.08
One	111516, 20.4 (2.6)		216605, 18.8 (1.8)		82764, 18.4 (2.9)	
Two or more	125462, 23.0 (3.2)		231112, 20.1 (2.4)		107973, 24.1 (3.3)	
Chronic conditions (type)						
Asthma	63471, 11.6 (2.3)	0.03	116685, 10.1 (1.5)	0.05	56158, 12.5 (2.6)	0.06
Depression	67957, 12.4 (2.5)		130053, 11.3 (1.7)		57944, 12.9 (3.0)	
Diabetes	42983, 7.9 (1.9)		81024, 7.0 (1.2)		39052, 8.7 (2.2)	
Hypertension	94341, 17.2 (2.9)		166277, 14.4 (1.8)		71729, 15.9 (2.8)	
Obesity	30821, 5.6 (1.8)		68407, 5.9 (1.9)		21807, 4.9 (1.7)	
SUD	54185, 9.9 (2.4)		97091, 8.4 (1.8)		42631, 9.4 (2.2)	
Primary diagnosis^b^						
Dental caries	102676, 18.8 (3.2)	<.0001	203183, 17.7 (2.1)	<.0001	85720, 19.1 (3.6)	<.0001
Pulpal and periapical	160275, 29.4 (3.2)		381310, 33.1 (2.3)		137911, 30.7 (3.4)	
Disorders of teeth	237014, 43.4 (3.4)		464674, 40.4 (2.6)		190654, 42.5 (3.6)	
Other:						
Tongue	1816, 0.3 (0.2)		10015, 0.9 (0.3)		0 (0)	
Cellulitis	5417, 1.0 (0.6)		15967, 1.4 (0.5)		5417, 1.2 (0.7)	
Periodontal disease	9746, 1.8 (1.1)		24013, 2.1 (1.1)		9746, 2.2 (1.4)	
Other	28772, 5.3 (1.7)		51948, 4.5 (0.9)		19489, 4.3 (1.6)	
Severe pain						
Yes	316000, 57.9 (4.3)	<.0001	555551, 48.3 (3.8)	<.0001	257515, 57.4 (5.0)	<.0001
No	229716, 42.1 (4.3)		595558, 51.7 (3.8)		191421, 42.6 (5.0)	
Fever						
Yes	1646, 0.3 (0.3)	0.005	19053, 1.7 (0.7)	0.25	1646, 0.4 (0.4)	0.02
No	544071, 99.7 (0.3)		1132056, 98.3 (0.7)		447290, 99.6 (0.4)	
Visit year						
2018	124354, 33.2 (3.5)	0.49	244205, 65.1 (3.0)	0.19	100069, 36.7 (3.2)	0.96
2019	131521, 31.4 (3.8)		250196, 59.8 (3.3)		101406, 24.2 (3.4)	
2020	102308, 31.6 (4.6)		225631, 69.7 (5.4)		77403, 23.9 (3.6)	
2021	90946, 26.6 (3.8)		218705, 63.9 (5.3)		80752, 23.6 (4.0)	
2022	96587, 25.4 (3.6)		212372, 55.9 (4.3)		89306, 23.5 (3.6)	

^a^ Type of chronic condition includes depression, diabetes, substance use disorder and other for associational analysis. *p* values calculated using chi‐squared testing.

^b^ Combined primary diagnosis into dental caries, pulpal, unspecified and other for associational analysis. *p* values calculated using chi‐squared testing.

Patient age, number of chronic conditions, severe pain, fever, and teaching hospital status were associated with an opioid prescription controlling for visit type (Table 4). Controlling for other factors, opioid prescriptions were less likely to occur at community hospitals (OR: 0.6, 95% CI 0.4–0.9, *p* = 0.02) and when the patient presented with an objective fever (OR: 0.07, 95% CI 0.007–0.8, *p* = 0.03) (Table 5). An opioid prescription was more likely when a patient presented with severe pain (OR: 2.1, 95% CI 1.5–2.9, *p* < 0.0001), or had a diagnosis of dental caries, pulpal and periapical disorders, or disorders of the teeth and supporting structures.

**TABLE 5:** Predictors of prescriptions among United States emergency department visits for NTDCs (2018–2022).

	Opioid	Antibiotic
Characteristics	aOR, 95% CI (*p* value)	
Age		
< 18	0.3, 0.06‐1.2 (0.09)	0.2, 0.08‐0.7 (0.009)
18‐39	1.3, 0.4‐3.6 (0.63)	0.6, 0.2‐1.8 (0.35)
40‐64	1.4, 0.5‐3.8 (0.56)	1.4, 0.5‐4.1 (0.57)
65+	Ref	Ref
Sex		
Male	Ref	Ref
Female	0.9, 0.7‐1.3 (0.70)	0.7, 0.5‐1.0 (0.04)
Race		
White	Ref	Ref
Non‐White	1.0, 0.6‐1.5 (0.82)	1.3, 0.8‐2.0 (0.22)
Ethnicity		
Hispanic or Latino	0.8, 0.5‐1.4 (0.41)	1.6, 0.9‐2.8 (0.07)
Non‐Hispanic or Latino	Ref	Ref
Primary payor		
Private	Ref	Ref
Medicare	0.6, 0.2–1.6 (0.31)	1.2, 0.5‐2.6 (0.71)
Medicaid	0.9, 0.6‐1.6 (0.75)	1.4, 0.8‐2.4 (0.29)
Self‐pay	0.9, 0.6‐1.6 (0.86)	1.5, 0.7‐3.0 (0.29)
Other or unknown	0.8, 0.4‐1.7 (0.58)	0.6, 0.3‐1.4 (0.24)
Provider type		
Physician	Ref	Ref
Non‐physician	0.9, 0.5–1.6 (0.71)	0.9, 0.6‐1.3 (0.58)
Hospital region		
Northeast	Ref	Ref
Midwest	1.1, 0.5‐2.5 (0.80)	1.4, 0.8‐2.6 (0.28)
West	1.7, 0.8‐3.8 (0.17)	1.2, 0.6‐2.2 (0.57)
South	1.3, 0.6‐2.5 (0.50)	0.8, 0.5‐1.5 (0.53)
Hospital rurality		
Urban	Ref	Ref
Rural	0.7, 0.3‐1.4 (0.34)	1.6, 0.8‐3.2 (0.21)
Hospital teaching status		
Academic	Ref	Ref
Community	0.6, 0.4‐0.9 (0.02)	0.5, 0.3‐0.7 (0.0003)
Blank or unknown	0.7, 0.4‐1.5 (0.47)	2.8, 0.8‐9.7 (0.11)
Chronic conditions (#)		
None	Ref	Ref
One	1.3, 0.8‐2.0 (0.25)	0.9, 0.5‐1.7 (0.83)
Two or More	1.7, 1.0‐2.9 (0.05)	1.1, 0.6‐1.9 (0.86)
Primary diagnosis		
Dental caries	3.3, 1.4‐7.5 (0.006)	11.7, 5.4‐25.6 (<.0001)
Pulpal and periapical	3.0, 1.5‐5.8 (0.002)	14.7‐7.4‐29.3 (<.0001)
Disorders of teeth	2.7, 1.4‐5.3 (0.004)	5.5, 3.2‐9.2 (<.0001)
Other	Ref	Ref
Severe pain		
Yes	2.1, 1.5‐2.9 (<.0001)	1.5, 1.0‐2.2 (0.08)
No	Ref	Ref
Fever		
Yes	0.07, 0.007‐0.8 (0.03)	0.7, 0.06‐6.8 (0.72)
No	Ref	Ref
Visit year		
2018	Ref	Ref
2019	1.0, 0.5‐1.7 (0.91)	0.7, 0.4‐1.3 (0.27)
2020	0.8, 0.4‐1.3 (0.31)	0.7, 0.4‐1.4 (0.33)
2021	0.8, 0.5‐1.4 (0.48)	1.3, 0.7‐2.5 (0.41)
2022	0.7, 0.4‐1.4 (0.30)	0.7, 0.4‐1.1 (0.08)
Disposition		
Admitted	0.7, 0.1‐4.2 (0.70)	1.4, 0.3‐5.4 (0.66)
D/c	Ref	Ref

*Note*: Caption: Model statistic for opioid (*c* = 0.72)—interaction between age and pain, sex and pain were not significant. Model statistic for antibiotics (*c* = 0.79)—interaction between age and pain, sex and pain were not significant.

### 3.3 Antibiotic Prescriptions for NTDCs

Overall, 62.6% (SE: 2.2) of NTDC visits included an antibiotic (Table 4). Penicillin antibiotics were the most common class prescribed (67.2%, SD: 2.1), followed by lincosamides (i.e., clindamycin) (28.1%, SD: 1.7). Overall, 40.% (SD: 2.6) of visits with an antibiotic had a disorder of the teeth and surrounding structures as the primary diagnosis, and 33.1% (SD: 2.3) had a diagnosis of a pulpal or periapical disorder (Table 4). Controlling for visit type, an antibiotic prescription was associated with patient age, pain, payor, teaching hospital status, and hospital rurality. Controlling for other factors, antibiotics were more likely for visits for pulpal and periapical disorders and less likely for pediatric patients, patients who were female, and visits that occurred at community hospitals (Table 5). About one‐quarter (24.4%, SD: 1.8) of NTDC visits resulted in both an antibiotic and an opioid (Table 4). Disorders of the teeth and surrounding structures (42.5%, SD: 3.6) and pulpal and periapical disorders (30.7%, SD: 3.4) were the most common primary diagnoses.

### 3.4 Prescriptions for NTDCs Over Time

Overall, there was no association between visit year and medication type observed, nor were there statistically significant between‐year differences in the proportion of medications prescribed (Tables 4 and 3). The proportion of NTDC visits with an opioid was highest in 2018 (33.2%, SE: 3.5), declining to 25.4% (SE: 3.6) in 2022, with a similar decline observed in concurrent prescriptions for antibiotics and opioids (Table 3). Non‐opioid analgesics and antibiotics were most commonly prescribed during the first year of the COVID‐19 pandemic (2020); 58.6% (SE: 5.1) included a non‐opioid, 69.7% (SE: 5.4) an antibiotic, and 45.1% (SE: 5.6) both a non‐opioid and an antibiotic, the highest proportions of each observed during the study period (Table 3).

## 4 Discussion

This study used nationally representative data to assess characteristics of and patterns among United States emergency department visits for non‐traumatic dental conditions that occurred between 2018 and 2022. Findings demonstrate a continued decline in NTDC visits resulting in an opioid with an increase in those with non‐opioids. Non‐opioid analgesics, in particular NSAIDs, have been shown to be as effective as, if not more, effective than opioids for management of inflammatory pain such as pulpitis, with fewer risks [13]. Antibiotics remained a common medication prescribed for NTDCs, consistent with prior studies [6, 7]. Increased reliance on medical management during the pandemic years (2020–2022) was also observed, with the highest proportion of non‐opioid analgesics and antibiotics seen in 2020. No increase in the proportion of ED visits for NTDCs was observed between the pandemic years compared to the pre‐pandemic period (2018 and 2019). Further, although there was an increase in the proportion of visits resulting in an admission observed in 2021, overall very few NTDC visits resulted in an admission to the hospital, suggesting that the majority of these visits remained low‐acuity.

Further reducing avoidable opioid and antibiotic prescriptions, and more broadly ED visits for NTDCs, requires a comprehensive approach. At the provider level, continued attention to optimal prescribing appears imperative. There are certain clinical situations in which opioids and/or antibiotics are indicated, and as such, prescriptions for these medications by EDs cannot be completely eliminated. Our findings suggest a shift toward non‐opioids for management of dental pain, with opioids more likely when patients present with severe pain. Antibiotics, although commonly prescribed, are also not without risk. For example, Wilson et al. demonstrated that the incidence of *Clostridioides difficile* following antibiotic prescription for dental problems was 50% in a small cohort [14]. In our study, antibiotics were more likely for diagnoses of pulpal and periapical disorders; however, it is unclear how many patients presented with systemic manifestations. Very few patients had an objective fever, while about 10% had a complete blood count or imaging ordered, and very few were admitted. Taken with the rise in antibiotics observed during the pandemic years, these findings suggest that the perceived availability (or lack thereof) of accessible outpatient oral healthcare services may be influencing prescribing practices [6]. Clinical practice guidelines for management of acute dental pain [15, 16] and use of antibiotics for odontogenic infections [17] were released between 2019 and 2024. These guidelines provide evidence‐based recommendations for when and how to prescribe medications for NTDCs. Clinical decision‐making tools embedded into electronic health record systems can also aid providers in managing NTDCs and have been shown to reduce unnecessary prescriptions [18]. Streamlining referrals to oral healthcare providers through the electronic health record in a manner commensurate with referrals to medical specialties may also be of benefit.

At the policy level, prior research suggests that ED visits for NTDCs are disproportionately made by working‐age adults, people from certain minoritized groups, and those with Medicaid or no medical insurance, patterns also seen in our study [19]. Financial barriers to accessing oral healthcare services are higher than for other health services [20, 21, 22] and dental insurance has shown to be protective against ED visits for NTDCs [23]. EDs typically do not have oral healthcare providers on staff who can help manage patients presenting with NTDCs, and increasing the availability of preventative and urgent oral healthcare services may be needed to reduce prescriptions [6]. Programs that divert patients presenting to EDs with NTDCs to established sites of definitive care (typically dental school clinics) have shown some success [24] although they have not been widely implemented. Ongoing partnerships between patients, private oral healthcare providers, health systems, and payors are likely needed to secure sustainable care for people presenting to EDs with NTDCs.

This study has several limitations. First, the unit of analysis for this study was a visit or encounter as opposed to the individual patient, in part due to the way in which the data were deidentified. Patients who presented more than once during the 4‐week hospital survey period may have been counted twice. It was assumed that the impact of this was small, although the impact cannot be ruled out. Second, the dataset did not allow for a consideration of duration of treatment or of filled versus unfilled prescriptions. Finally, whether or not the potency of prescribed opioids changed was not assessed using this dataset; however, available research suggests that the morphine equivalent dose of opioids prescribed by EDs during the early years of the COVID‐19 pandemic did increase [11]. Despite limitations, this study offers a nationally representative view of ED visits for NTDCs during the pandemic years (2020–2022) that could be used to direct future studies and pilot interventions.

## 5 Conclusions

In summary, this study demonstrates a decline in prescriptions for opioids among ED visits for non‐traumatic dental conditions through the COVID‐19 pandemic years of 2020–2022. There was a concurrent increase in the proportion of visits with non‐opioid analgesics. Antibiotics remain commonly prescribed by EDs for NTDCs. Further reducing avoidable opioid and antibiotic prescriptions, and more broadly ED visits for NTDCs, requires a comprehensive approach.

## Conflicts of Interest

The authors declare no conflicts of interest.

## Supporting information


**Data S1.** Supporting Information.

## Data Availability

These data were derived from the following resources available in the public domain: National Hospital Ambulatory Medical Care Survey, https://www.cdc.gov/nchs/nhamcs/documentation/index.html.
