# Supplementary material for: Pharmacologic Management of Non‐Traumatic Dental Conditions in US Emergency Departments, 2018–2022
Source: J Public Health Dent. 2025 Apr 11;85(3):220–30. doi: 10.1111/jphd.12668 (PMC12354039; doi:10.1111/jphd.12668)
Supplement: Supplementary file 1 — Data S1. Supporting Information. [file JPHD-85-220-s001.docx]

**SUPPLEMENTARY TABLES & FIGURES**

**Supplementary Table 1: ASTDD Codes for NTDCs (1)**

| ICD-10 Codes | Weighted Frequency (Unweighted) | % of Total NTDCs (SE) |
| --- | --- | --- |
| *Infectious & Parasitic Disorders*  A690, A691, B002, B084, B0861, B370, B3783 | 143892 (78) | 7.8 (1.2) |
| *Disorders of the Digestive System*  K00, K000, K001, K002, K003, K004, K005, K006, K007, K008, K009, K01, K010, K011, K02, K023, K025, K0251, K0252, K0253, K026, K0261, K0262, K0263, K027, K029, K03, K030, K031, K032, K033, K034, K035, K036, K037, K038, K0381, K0389, K039, K04, K040, K0401, K0402, K041, K042, K043, K044, K045, K046, K047, K048, K049, K0490, K0499, K05, K050, K0500, K0501, K051, K0510, K0511, K052, K0520, K0521, K05211, K05212, K05213, K05219, K0522, K05221, K05222, K05223, K05229, K053, K0530, K0531, K05311, K05312, K05313, K05319, K0532, K05321, K05322, K05323, K05329, K054, K0540, K055, K056, K06, K060, K0601, K06010, K06011, K06012, K06013, K0602, K06020, K06021, K06022, K06023, K061, K063, K068, K069, K08, K080, K081, K0810, K08101, K08102, K08103, K08104, K08109, K0812, K08121, K08122, K08123, K08124, K08129, K0813, K08131, K08132, K08133, K08134, K08139, K0819, K08191, K08192, K08193, K08194, K08199, K082, K0820, K0821, K0822, K0823, K0824, K0825, K0826, K083, K084, K0840, K08401, K08402, K08403, K08404, K08409, K0842, K08421, K08422, K08423, K08424, K08429, K0843, K08431, K08432, K08433, K08434, K08439, K0849, K08491, K08492, K08493, K08494, K08499, K0850, K0851, K0852, K0853, K08530, K08531, K08539, K0854, K0855, K0856, K0859, K088, K0889, K089, K09, K090, K091, K098, K099, K11, K110, K111, K112, K1120, K1121, K1122, K1123, K113, K114, K115, K116, K117, K118, K119, K12, K120, K121, K122, K123, K1230, K1231, K1232, K1233, K1239, K13, K130, K131, K132, K1321, K1322, K1323, K1324, K1329, K133, K134, K135, K136, K137, K1370, K1379, K14, K140, K141, K142, K143, K144, K145, K146, K148, K149 | 1653175 (1021) | 89.9 (1.2) |
| *Musculoskeletal & Connective Tissue Disorders*  M26, M260, M2600, M2601, M2602, M2603, M2604, M2605, M2606, M2607, M2609, M261, M2610, M2611, M2612, M2619, M262, M2620, M2621, M26211, M26212, M26213, M26219, M2622, M26220, M26221, M2623, M2624, M2625, M2629, M263, M2630, M2631, M2632, M2633, M2634, M2635, M2636, M2637, M2639, M264, M265, M2650, M2651, M2652, M2653, M2654, M2655, M2656, M2657, M2659, M266, M2660, M26601, M26602, M26603, M26609, M2661, M26611, M26612, M26613, M26619, M2662, M26621, M26622, M26623, M26629, M2663, M26631, M26632, M26633, M26639, M2664, M26641, M26642, M26643, M26649, M2665, M26651, M26652, M26653, M26659, M2669, M267, M2670, M2671, M2672, M2673, M2674, M2679, M268, M2681, M2682, M2689, M269, M27, M270, M271, M272, M273, M274, M2740, M2749, M275, M2751, M2752, M2753, M2759, M276, M2761, M2762, M2763, M2769, M278, M279, M350C, M7911 | 39330 (26) | 2.1 (0.5) |
| *Symptoms (i.e. Subjective Dry Mouth, Jaw Pain)*  R682, R6884 | 1407 (3) | 0.08 (0.05) |
| *Encounter Codes*  Z012, Z0120, Z0121, Z463, Z464 | 924 (1) | 0.05 (0.05) |

**Supplementary Table 2: NTDC Category Codes (ICD-10)**

| ICD-10 Codes | Weighted Frequency (Unweighted) | % of Total NTDCs (SE) |
| --- | --- | --- |
| *Dental Caries*  K02, K023, K025, K0251, K0252, K0253, K026, K0261, K0262, K0263, K027, K029 | 244969 (141) | 13.3 (1.6) |
| *Pulpal and Periapical Disorders*  K04, K040, K0401, K0402, K041, K042, K043, K044, K045, K046, K047, K048, K049, K0490, K0499 | 463097 (281) | 25.2 (1.7) |
| *Disorders of Teeth and Surrounding Structures*  K08, K080, K081, K0810, K08101, K08102, K08103, K08104, K08109, K0812, K08121, K08122, K08123, K08124, K08129, K0813, K08131, K08132, K08133, K08134, K08139, K0819, K08191, K08192, K08193, K08194, K08199, K082, K0820, K0821, K0822, K0823, K0824, K0825, K0826, K083, K084, K0840, K08401, K08402, K08403, K08404, K08409, K0842, K08421, K08422, K08423, K08424, K08429, K0843, K08431, K08432, K08433, K08434, K08439, K0849, K08491, K08492, K08493, K08494, K08499, K0850, K0851, K0852, K0853, K08530, K08531, K08539, K0854, K0855, K0856, K0859, K088, K0889, K089 | 688896 (429) | 37.5 (2.1) |
| *Disorders of the Tongue*  K13, K130, K131, K132, K1321, K1322, K1323, K1324, K1329, K133, K134, K135, K136, K137, K1370, K1379, K14, K140, K141, K142, K143, K144, K145, K146, K148, K149 | 96052 (60) | 5.2 (0.9) |
| *Cellulitis and Abscess*  K122 | 23284 (19) | 1.3 (0.4) |
| *Periodontal Disorders*  K05, K050, K0500, K0501, K051, K0510, K0511, K052, K0520, K0521, K05211, K05212, K05213, K05219, K0522, K05221, K05222, K05223, K05229, K053, K0530, K0531, K05311, K05312, K05313, K05319, K0532, K05321, K05322, K05323, K05329, K06, K060, K0601, K06010, K06011, K06012, K06013, K0602, K06020, K06021, K06022, K06023, K061, K063, K068, K069 | 42300 (24) | 2.3 (0.8) |
| *Other Disorders^1^*  K03, K038, K0389, K039, K11, K110, K111, K112, K1120, K1121, K1122, K1123, K113, K114, K115, K116, K117, K118, K119, K12, K120, K121, K123, K1230, K1231, K1232, K1233, K1239, A690, A691, B002, B084, B0861, B370, B3783, M26, M260, M2600, M2601, M2602, M2603, M2604, M2605, M2606, M2607, M2609, M261, M2610, M2611, M2612, M2619, M262, M2620, M2621, M26211, M26212, M26213, M26219, M2622, M26220, M26221, M2623, M2624, M2625, M2629, M263, M2630, M2631, M2632, M2633, M2634, M2635, M2636, M2637, M2639, M264, M265, M2650, M2651, M2652, M2653, M2654, M2655, M2656, M2657, M2659, M266, M2660, M26601, M26602, M26603, M26609, M2661, M26611, M26612, M26613, M26619, M2662, M26621, M26622, M26623, M26629, M2663, M26631, M26632, M26633, M26639, M2664, M26641, M26642, M26643, M26649, M2665, M26651, M26652, M26653, M26659, M2669, M267, M2670, M2671, M2672, M2673, M2674, M2679, M268, M2681, M2682, M2689, M269, M27, M270, M271, M272, M273, M274, M2740, M2749, M275, M2751, M2752, M2753, M2759, M276, M2761, M2762, M2763, M2769, M278, M279, M350C, M7911, R682, R6884, Z012, Z0120, Z0121, Z463, Z464 | 280132 (175) | 15.2 (1.4) |

^1^ Includes salivary gland disorders, cysts, musculoskeletal disorders, encounter codes, stomatitis, and infectious disorders such as candidiasis.

**Supplementary Table 3: Multum Codes for Analgesics and Antibiotics**

| Medication Type | Multum Codes |
| --- | --- |
| *Opioid* | 060, 191 |
| *Non-Opioid* | 059, 061, 062, 063, 278 |
| *Antibiotic* | 006, 008, 009, 010, 011, 012, 013, 014, 015, 016, 017, 018, 240, 315, 406, 486, 499, 503 |

**Supplementary Table 4: Characteristics of Hospital Admissions for NTDCs (United States, 2018-2022)**

| Characteristic | Unweighted Freq.  n= 17 | Weighted Freq.  n= 32514 | % (SE) |
| --- | --- | --- | --- |
| *Age*  < 18  18-39  40-64  65+ | 3  6  2  6 | 8248  17510  2887  3869 | 25.4 (14.5)  53.9 (14.9)  8.9 (7.1)  11.9 (6.2) |
| *Sex*  Male  Female | 8  9 | 12399  20114 | 38.1 (13.3)  61.9 (13.3) |
| *Race*  African American/Black  White  Other | 7  9  1 | 14056  18278  179 | 43.2 (17.2)  56.2 (17.2)  0.6 (0.6) |
| *Ethnicity*  Hispanic or Latino  Non-Hispanic or Latino | 1  16 | 5369  27144 | 16.5 (14.7)  83.5 (14.7) |
| *Primary Payor*  Private  Medicare  Medicaid  Self-Pay  Other or Unknown | 4  5  3  0  5 | 5714  3626  7540  0  15633 | 17.6 (9.8)  11.2 (6.1)  23.2 (11.4)  0 (0)  48.1 (17.2) |
| *Hospital Region*  Northeast  Midwest  West  South | 3  7  2  5 | 1357  14131  6039  10989 | 4.2 (2.9)  43.5 (16.7)  18.5 (15.0)  33.7 (16.4) |
| *Hospital Rurality*  Urban  Rural | 17  0 | 32514  0 | 100.0 (0)  0 (0) |
| *Hospital Teaching Status*  Academic  Community | 9  8 | 16958  15555 | 52.2 (15.0)  47.8 (15.0) |
| *Chronic Conditions (#)*  None  One  Two or More | 6  4  7 | 12685  9138  10691 | 39.0 (15.0)  28.1 (15.7)  32.9 (12.4) |
| *Chronic Condition (Type)*  Asthma  Depression  Diabetes  Hypertension  Obesity  Substance Use Disorder | 1  1  3  7  2  0 | 2660  2937  4236  11381  5303  0 | 8.2 (8.0)  9.0 (7.9)  13.1 (8.3)  35.0 (16.0)  16.3 (10.0)  0 (0) |
| *Severe Pain*  Yes  No | 3  14 | 3990  28523 | 12.2 (8.6)  87.8 (8.6) |
| *Fever*  Yes  No | 2  15 | 2850  29663 | 8.7 (7.4)  91.2 (7.4) |
| *Primary Diagnosis*  Dental Caries  Pulpal and Periapical  Disorders of Teeth  Disorders of the Tongue  Cellulitis  Periodontal Disease  Other | 0  6  0  1  4  0  6 | 0  20288  0  1196  5991  0  5039 | 0 (0)  62.4 (14.5)  0 (0)  3.7 (3.7)  18.4 (10.8)  0 (0)  15.5 (8.8) |
| *Length of Stay (Days)*  Median  Range | 3  1-5 | -  - | -  - |
| *Provider Type*  Physician  Non-Physician | 15  2 | 31538  976 | 97.0 (2.5)  3.0 (2.5) |
| *Visit Year*  2018  2019  2020  2021  2022 | 2  3  1  5  6 | 623  11802  2937  10818  6333 | 0.17 (0.1)  2.8 (1.7)  0.9 (0.9)  3.2 (1.8)  1.7 (0.9) |

**Supplementary Table 5: Characteristics of Teaching Hospitals Where NTDCs Seen (United States, 2018-2022)**

|  | Teaching |  | Non-Teaching |  | | p value | |
| --- | --- | --- | --- | --- | --- | --- | --- |
| Characteristic | Weighted Freq | % (SE) | Weighted Freq | % (SE) | | |  |
| *Region*  Northeast  Midwest  West  South | 99432  180418  336820  581213 | 8.3 (2.5)  15.1 (2.6)  28.1 (3.9)  48.5 (5.4) | 175527  205655  70098  103065 | 31.7 (6.9)  37.1 (6.3)  12.6 (3.5)  18.6 (4.5) | | <.0001 | |
| *Provider Type*  Physician  Non-Physician | 847889  349993 | 70.8 (3.3)  29.2 (3.3) | 428518  125826 | 77.3 (4.9)  22.7 (4.9) | 0.24 | | |
| *Primary Diagnosis*  Dental Caries  Pulpal/Periapical  Disorders of Teeth  Other | 189455  315753  424455  265345 | 15.9 (1.9)  26.4 (2.2)  35.5 (2.2)  22.2 (2.0) | 40985  127900  219157  165894 | 7.4 (1.9)  23.1 (2.5)  39.6 (3.7)  29.9 (3.5) | 0.002 | | |
| *Severe Pain*  Yes  No | 3933492  83620701 | 4.5 (0.2)  95.5 (0.2) | 2429346  42414104 | 5.5 (0.4)  94.6 (0.4) | 0.14 | | |
| *Fever*  Yes  No | 32711  1165170 | 2.7 (1.0)  97.2 (1.0) | 6914  547431 | 1.3 (0.6)  98.8 (0.6) | 0.34 | | |
| *Disposition*  Admitted  Discharged | 16958  1180923 | 1.4 (0.6)  98.6 (0.6) | 15555  625293 | 2.4 (1.1)  97.6 (1.1) | 0.36 | | |

**Supplementary Table 6: Characteristics of Patients Presenting with Severe Pain (United States, 2018-2022)**

|  | Severe Pain |  | No Severe Pain |  | | p value | |
| --- | --- | --- | --- | --- | --- | --- | --- |
| Characteristic | Weighted Freq | % (SE) | Weighted Freq | % (SE) | | |  |
| *Age*  < 18  18-39  40-64  65+ | 23641  474119  204174  31311 | 3.2 (1.3)  64.7 (3.0)  27.8 (2.5)  4.3 (1.2) | 293238  524794  232394  55059 | 26.5 (2.7)  47.5 (2.7)  21.0 (1.8)  5.0 (1.2) | | <.0001 | |
| *Sex*  Male  Female | 338371  394873 | 46.1 (2.8)  53.9 (2.8) | 534263  571222 | 48.3 (2.2)  51.7 (2.2) | 0.51 | | |
| *Race*  AA/Black  White  Other | 223652  497306  12286 | 30.5 (3.3)  67.8 (3.3)  1.7 (0.9) | 369241  716868  19376 | 33.4 (2.9)  64.8 (3.0)  1.8 (0.6) | 0.75 | | |
| *Ethnicity*  Hispanic or Latino  Non-Hispanic or Latino | 96601  636644 | 13.2 (2.7)  86.8 (2.7) | 170924  934561 | 15.5 (2.3)  84.5 (2.3) | 0.48 | | |
| *Primary Payor*  Private  Medicare  Medicaid  Self-Pay  Other or Unknown | 139850  50854  381074  113799  47668 | 19.1 (2.2)  6.9 (1.6)  51.9 (3.4)  15.5 (2.2)  6.5 (1.5) | 230484  80025  510072  128450  156456 | 20.8 (2.4)  7.2 (1.4)  46.1 (4.3)  11.6 (1.8)  14.2 (3.8) | 0.04 | | |
| *Primary Diagnosis*  Dental Caries  Pulpal/Periapical  Disorders of Teeth  Other | 109212  207250  346054  68668 | 14.9 (2.2)  28.3 (2.8)  47.3 (3.4)  9.4 (1.9) | 135756  255847  342842  369819 | 12.3 (1.9)  23.2 (1.7)  31.0 (1.9)  33.5 (2.3) | <.0001 | | |
